# The Effect of *Lactiplantibacillus plantarum* x3-2b Bacterial Powder on the Physicochemical Quality and Biogenic Amines of Fermented Lamb Jerky

**DOI:** 10.3390/foods12224147

**Published:** 2023-11-16

**Authors:** Xiaotong Li, Guanhua Hu, Xueying Sun, Erke Sun, Yue Zhang, Yancheng Zhong, Lin Su, Ye Jin, Fan Yang, Lihua Zhao

**Affiliations:** 1 Department of Food Science, College of Food Science and Engineering, Inner Mongolia Agricultural University, Hohhot 010018, China; 18748342389@163.com (X.L.); 18247109171@163.com (G.H.); m15774711156@163.com (X.S.); sun15848107303@163.com (E.S.); zhangyueimau@163.com (Y.Z.); sulin820911@163.com (L.S.); jinyeyc@sohu.com (Y.J.); 2 Beijing Tongzhou District Health Commission, Beijing 101100, China; 17310261312@163.com; 3 Ordos Vocational College of Eco-Environment, Kangbashi District, Ordos 017010, China

**Keywords:** functional starter, protective agent, fermented meat product, physicochemical quality, safety

## Abstract

In this study, a protective agent was added to prepare a high-activity *Lactiplantibacillus plantarum* x3-2b bacterial powder as a fermentation agent and explore its effect on the physicochemical quality, biogenic amines, and flavor of fermented lamb jerky. A composite protective agent, composed of 15% skim milk powder and 10% trehalose, was used, and bacterial mud was mixed with the protective agent at a 1:1.2 mass ratio. The resulting freeze-dried bacterial powder achieved a viable count of 5.1 lg CFU/g with a lyophilization survival rate of 87.58%. Scanning electron microscopy revealed enhanced cell coverage by the composite protective agent, maintaining the cell membrane’s integrity. Inoculation with x3-2b bacterial powder increased the pH and the reduction in a_w_, enhanced the appearance and texture of fermented lamb jerky, increased the variety and quantity of flavor compounds, and reduced the accumulation of biogenic amines (phenethylamine, histamine, and putrescine). This research provides a theoretical basis for improving and regulating the quality of lamb jerky and establishes a foundation for the development of bacterial powder for the commercial fermentation of meat products.

## 1. Introduction

Fermented lamb jerky is defined as a fermented meat product crafted by harnessing microbial activity under natural or artificially controlled conditions, which then undergoes processes including marination, fermentation, drying, maturation, or roasting. It has many advantages, such as high nutritional value, an attractive color, and a prolonged shelf life. However, the traditional processing of fermented meat jerky is hindered by uncontrollable natural factors such as wind speed, temperature, and humidity, resulting in quality issues such as poor texture and color [1,2,3]. Additionally, it is prone to the formation of high concentrations of harmful substances, such as biogenic amines (BAs) [4]. BAs are substances formed through the decarboxylation of amino acids by microbially secreted amino acid decarboxylases during the fermentation process. The accumulation of BAs in the final product is influenced by specific microbial strains and fermentation conditions. Consequently, the exploration of methods to reduce the content of BAs in fermented meat jerky while simultaneously enhancing the overall quality of the product is of paramount significance.

With the application and popularization of modern fermentation technology, artificial directional inoculation has a functional starter for improving the quality of fermented meat jerky, which may not only overcome the problems of the traditional process such as poor tenderness, low yield, and dark color, but may also optimize the flavor, color and texture of the product. *Lactic acid bacteria* (LAB) hold the distinction of being the most extensively utilized fermentation agents in the realm of fermented meat products. The fermentation of raw meat through the metabolism of LAB and endogenous enzymes induces a cascade of physiological and biochemical reactions, thereby enhancing attributes such as the flavor, color, texture, and safety of fermented meat products. A plethora of studies have indicated that the formation of flavor precursors such as amino acids, esters, peptides, and short-chain fatty acids in fermented meat products is associated with the characteristics of LAB [5,6]. Concurrently, LAB generate bacteriocins, including substances such as nisin, which serve as antimicrobial agents. These compounds inhibit the growth of pathogens such as *Listeria* and *Staphylococcus aureus*, preventing spoilage in fermented meat products [7]. LAB fermentation also hinders the growth of microorganisms with amino acid decarboxylation capabilities, preventing the accumulation of BAs in fermented meat products and consequently enhancing their safety. For example, Sun et al. [8] verified that a compound fermentation agent of *Staphylococcus xylosus* and *Ligilactobacillus salivarius* could effectively inhibit the accumulation of BAs in Harbin air-dried sausage. Zhang et al. [9] found that the amine oxidase produced by adding *Lactiplantibacillus plantarum* and *Lactobacillus salivarius* to traditional smoked horsemeat sausage had the effect of degrading BAs.

As the use of LAB fermentation agents in the production of meat products continues to expand, preservation techniques have garnered widespread attention. Liquid and semi-solid preservation methods are more prevalent preservation technologies, but there are defects, such as the easy depletion of bacteria, the ease of pollution, the need for a strict controlled storage temperature, and its inconvenient storage and transportation. These limitations hinder the large-scale production of LAB fermentation agents and fermented products. In contrast, bacterial powders enable industrial-scale production, and facilitate transportation and storage [10]. Freeze-drying is one of the more common techniques used in the production of bacterial powder. During vacuum freeze-drying, protective agents are usually added to avoid different degrees of damage to the cells, thereby improving the cells’ survival rate. Research by Abadias et al. [11] found that combining 10% skim milk with other protective agents (such as 5% or 10% glucose, or 10% fructose or sucrose) as a composite protective formulation increased yeast cells’ viability from 0.2% to 30–40%. Similarly, the study by Ming et al. [12] revealed that in freeze-dried, using skim milk and sucrose alone as protective agents was more conducive to preserving *Lactobacillus salivarius* I 24 compared with glycerol and calcium carbonate. Notably, when used as a mixture (9.85% *w*/*v* skim milk and 10.65% *w*/*v* sucrose), the effect was significantly enhanced. It can be seen that the protective effect of the protective agent on the bacteria changes with the type of microorganism. Therefore, it is particularly important to select an appropriate protective agent according to the specificity of the strain [13].

This study investigated the effects of different formulations of protective agents on the lyophilization survival rate of *Ligilactobacillus plantarum* (x3-2b) bacteria powder, and the effects of x3-2b bacterial powder on the physicochemical properties, BAs, and flavor during the processing of fermented lamb jerky. The findings of this study offer a theoretical foundation for enhancing the quality and ensuring the safety of fermented lamb jerky.

## 2. Materials and Methods

### 2.1. Raw Materials and Reagents

To obtain the meat, 6-month-old sunit sheep (males, with a carcass weight of about 15 kg) were selected. Fresh hind leg lamb meat with 24 h of post-slaughter acid removal was provided by Inner Mongolia Grassland Jingxin Food Co., Ltd., (Bayan Nur, Inner Mongolia, China) as raw material. The skim milk powder was provided by Inner Mongolia Yili Industrial Co., Ltd., (Hohhot, Inner Mongolia, China). *Lactiplantibacillus plantarum* x3-2b was provided by the Meat Science and Technology Team (School of Food Science and Engineering, Inner Mongolia Agricultural University, Hohhot, China). Trehalose was purchased from Japan Co., Ltd., (Okayama City, Japan). Sodium nitrite and p-aminobenzene sulfonic acid were purchased from National Pharmaceutical Group Chemical Reagents Co., Ltd., (Beijing, China). 2-methyl-3-heptanone, dansyl chloride and BAs were purchased from Sigma Aldrich (St. Louis, MO, USA). All other reagents were purchased from Sinopharm Chemical Reagent Company Limited (Beijing, China).

### 2.2. Determination of the Viable Counts and Lyophilization Survival Rates of the Strains

The x3-2b strain was activated for three generations and cultured in MRS medium for 48 h. The viable count of the third-generation strain at different time points (0, 2, 4, 6, 8, 12, 24, and 36 h) was determined according to the plate colony-counting technique, and three repeated experiments were carried out [14].

The samples of bacterial fluids were pre-frozen at −80 °C for 24 h, then vacuum freeze-dried at −80 °C for 24 h using a vacuum freeze-dryer (Alpha 1–4 LSC Baisc, Marin Christ, Osterode, Germany), and then placed into aluminum foil pouches, which were vacuum-sealed and preserved at −80 °C. The freeze-dried bacterial powder was rehydrated with 0.1 mol/L of a phosphate-buffered solution at 37 °C added to the pre-freeze-dried volume [15]. Then, it and the original bacterial solution before freeze-drying were, respectively inoculated on the MRS medium at the inoculum rate of 2%. Subsequently, the viable count was measured via the following formula [16]:

$$\mathrm{Lyophilization}\;\mathrm{survival}\;\mathrm{rate}\;(\%)=N_{0}/N\times 100\%$$

where N_0_ is the viable count after the lyophilized bacterial powder was rehydrated to the volume of the original bacterial solution (CFU/mL), and N is the number of viable bacteria per unit of volume of the original bacterial solution before freeze-drying.

### 2.3. Preparation of the x3-2b Bacteria Powder

The x3-2b strain was activated for three generations on the MRS medium. With the viable counts and lyophilization survival rate as indicators, skim milk powder (5%, 10%, 15%, 20%, and 25%) was added. After sterilization at 121 °C for 15 min, the sample was mixed with bacterial mud at a 1:1 (*w*/*w*) ratio and freeze-dried to determine the optimal amount of skim milk powder in the composite protective agent. Trehalose (5%, 10%, 15%, 20%, and 25%) was added to the skim milk solution to create the composite protective agent. The bacterial mud was mixed with the protective agent at various mass ratios (1:0.3, 1:0.6, 1:0.9, 1:1.2, and 1:1.5) and then freeze-dried for preservation [17].

### 2.4. Scanning Electron Microscopy (SEM)

In line with the approach of Divan et al. [18], the bacterial powder was affixed to the sample stage using a conductive adhesive under a vacuum after sputter-coating with gold at an acceleration voltage of 15 kV and a working distance of 6-7 mm, and observed using an electron microscope (S-3400N, Hitachi Ltd., Tokyo, Japan).

### 2.5. Development of Fermented Lamb Jerky

This experiment used fresh hind leg lamb meat as raw material (removed tendons and subcutaneous fat, and then cut into 4 cm × 2 cm × 1 cm strips along the muscle fibers) to independently produce three batches of fermented lamb jerky. For each batch, 3 kg of hind leg lamb meat was taken and divided equally into three groups. The lamb jerky that was not inoculated with the fermentation agent served as the control group (CO), alongside the group with a single protection agent (skim milk powder (SP)) and the group with the composite protection agent (skim milk powder and trehalose (CP)). Each group was produced by the same process formula. The actual amount of additives added per 1 kg of ingredients was 5.0 g of sucrose, 1.0 g of glucose, 25.0 g of NaCl, 0.002 g of sodium nitrite, 5.0 g of white pepper powder, 5.0 g of ginger powder, and 2.0 g of L-ascorbic acid. The starter culture of 2% x3-2b bacterial powder (10^7^ CFU/g meat) was injected into the upper, middle and bottom three sections of the cut meat strips and marinated for 12 h. Then, fermentation was carried out for 24 h at 95% relative humidity and 30 °C, followed by baking at 90 °C for 2 h.

### 2.6. Determination of the Physical and Chemical Indicators of Fermented Lamb Jerky

The determination of the pH and water activity (a_w_) values followed the method of Chen et al. [19] with slight modifications. Five grams of minced fermented lamb jerky was homogenized with 45 mL of physiological saline in a blender. The pH was measured using an electronic pH meter (Mettler Toledo Instruments Co., Ltd., Shanghai, China). The fermented lamb jerky particles were evenly spread on the bottom of a measurement dish, and the a_w_ value was measured using an automatic water activity meter (WA-160A, Decagon, Pullman, WA, USA). A TPC automatic colorimeter (Olympus Optics, Beijing, China) was used to measure the differences in color from the reference E* values for analysis and evaluation, calculated by the formula: E* = a*/L* + a*/b* [3].

According to the method of Luo et al. [20], the fermented lamb jerky was cut into l cm × l cm × l cm cubes. A QTS25 texture analyzer (Xiamen Chaoji Instrument Co., Ltd., Xiamen, China) with a T-type probe (p/5) was used to measure the hardness, chewiness, elasticity, cohesion, stickiness, and resilience of the jerky when compressed to 50% of its height. The parameters of the texture analyzer were set as follows: pre test speed: 2.0 mm/s; test speed: 1.0 mm/s; return speed: 2.0 mm/s; number of cycles: 2; and time interval between two presses: 5.0 s. Each test was repeated 3 times. The determination of the thiobarbiturate acid (TBARS) followed the method of Sun et al. [3], with slight modifications. Approximately 4.0 g of fermented meat jerky was mixed with 20 mL of 75% trichloroacetic acid (containing 0.1% EDTA) and shaken for 30 min. After filtering twice through a double layer of filter paper, 5 mL of the supernatant was collected, 5 mL of 0.02 mol/L TBARS solution was added, and the solution was kept in a water bath at 90 °C for 40 min. The supernatant was then centrifuged at 453× *g* for 5 min. Next, 8 mL of the supernatant was added to 5 mL of chloroform, shaken well and allowed to stand. A SYNERGY multifunctional microplate reader (American BIOTEK Co., Ltd., Winooski, VT, USA) was used to measure the absorbance at 532 nm and 600 nm. The nitrite residue was quantified according to the method of GB5009.33-2016 [21].

### 2.7. Determination of Biogenic Amines (BAs)

According to the method of Lu et al. [22], a 2.0 g sample was mixed with 20 mL of 0.4 M perchloric acid, diluted to 50 mL with 0.4 M perchloric acid, and centrifuged at 5000× *g* for 10 min at 4 °C. Next, 1 mL of the sample extract was derivatized with 200 μL of NaOH (2 M), 300 μL of Na_2_CO_3_ (saturated), and 2 mL of dansyl chloride (10 mg/mL). After derivatization, 10 μL of each sample filtrate was filtered with a 0.22 μm filter membrane and injected into a chromatographic column (ZORBAX SB-C18: 250 mm × 4.6 mm; particle size, 5 μL; Agilent). The column was filled with the sample at a flow rate of 0.9 mL/min with an ultraviolet detection wavelength of 254 nm. High-performance liquid chromatography (HPLC) analysis was performed (Agilent 1260, Agilent, Santa Clara, CA, USA). The parameters during the 0–5 min period were as follows: Mobile Phase A (acetonitrile), 35–25%; and Mobile Phase B (water), 65–75%. Over 44–55 min, the proportions were as follows: Mobile Phase A, 35%; and Mobile Phase B, 65%. The isolated BAs were identified and compared to the retention time of the known standard (1,7-diaminoheptane).

### 2.8. Determination of Volatile Flavor Substances

In line with Wen et al.’s method [23], the volatile flavor compounds in the fermented lamb jerky were extracted by solid phase microextraction (SPME) and determined by a gas chromatography–mass spectrometry (GC/MS) system (GCMS-QP2020 NX, Shimadzu Corporation, Kyoto, Japan) [19]. We weighed 5.0 g of the fermented lamb jerky and placed it in a bottle with a 20 mL headspace, and 5 mL saturated sodium chloride solution and 1 μL of an internal standard of 0.168 μg/μL 2-methyl-3-heptanone was added and mixed. The mixture was placed in a rotor and oscillated on a magnetic stirrer. The aged SPME extraction head was inserted into the headspace of the sample bottle, and the sample was adsorbed in a water bath at 60 °C for 30 min. After adsorption, it was taken out and inserted into the inlet of the gas chromatography, and resolved at 250 °C for 3 min. The gas chromatography conditions were as follows: a DB capillary column (30 m × 0.25 mm × 0.25 μm); helium as the carrier gas at a flow rate of 1 mL/min; an injection port interface temperature of 250 °C; the temperature program: the initial temperature was 40 °C, kept for 3 min, increased to 230 °C at 4 °C/min, kept for 5 min; a splitless mode of injection; ion source temperature 250 °C; transmission line temperature 250 °C; the mass scanning range was *m*/*z* 20~300; the solvent was delayed for 1 min. The mass spectrum was qualitatively searched with MEANLIB, NistDemo, and Wiley Library, and the matching degree was greater than 800 as the basis for identification. 2-methyl-3-heptanone was selected as the internal standard to determine volatile flavor substances, and quantification was performed based on the peak area of 2-methyl-3-heptanone with a known mass concentration. 

### 2.9. Statistical Analysis

All experimental measurements were independently repeated three times, and the results were expressed as the mean ± standard error (SE). The results were analyzed using the SPSS statistical package for Windows V26.0 (IBM, Chicago, IL, USA). Single factor analysis of variance (ANOVA) and the LSD test (as a post hoc test) were performed. The results were considered to be statistically significant at *p* < 0.05. The charts in this article were made using Origin 2019 (Origin Lab Inc., Northampton, MA, USA).

## 3. Results and Discussion

### 3.1. Optimization of the Added Amount of Freeze-Dried Protective Agent

**Figure 1:**
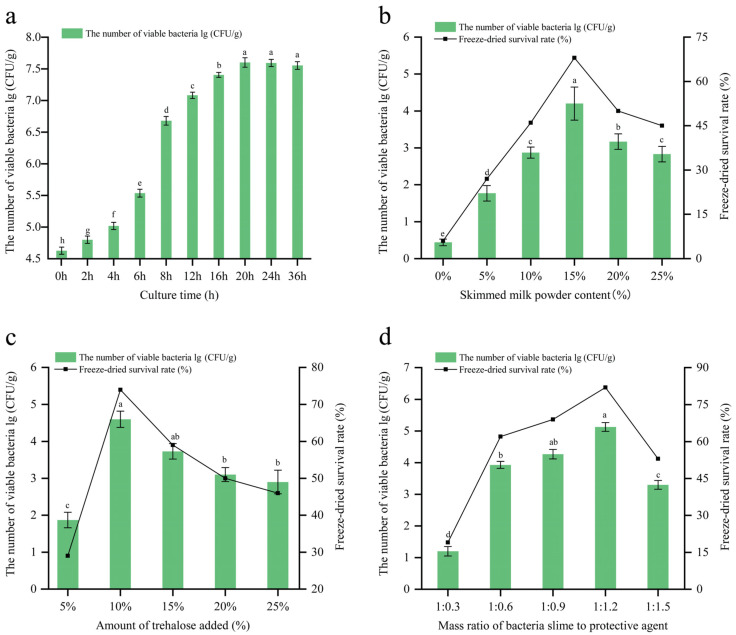
Viable bacteria of strain x3-2b at different incubation times (**a**). Effect of different protective agent additions on the number of viable bacteria and freeze-dried survival of strain x3-2b ((**b**), milk powder; (**c**), trehalose; (**d**), mass ratio of bacteria slime to protective agent). Different letters (a–h) indicate significant differences at *p* < 0.05.

As shown in Figure 1a, the x3-2b strain grew rapidly within 4 to 8 h, entered the logarithmic growth phase, and stabilized after 20 h (7.63 lg CFU/g). A decrease in the viable count occurred at 36 h, possibly because the mortality rate of the strain exceeded the division rate, and it began to enter the period of decay, but the difference was not significant compared with the count at 24 h, which proved that the strain had an active cell population and a high viable count and was conducive to the formulation of freeze-dried powder. 

The proteins in skim milk powder can form a protective layer outside cells, isolating the strains from oxygen and preventing damage during the freeze-drying process. As shown in Figure 1b, with an increase in the content of skim milk powder, both the viable count and the lyophilization survival rate of the strains exhibited an upward trend. When the content of skim milk powder reached 15%, the viable count (4.2 lg CFU/g) and lyophilization survival rate (68%) reached their maximum values (*p* < 0.05). This indicated that the addition of 15% skim milk powder enabled full interaction with the bacterial cells, effectively protecting them [24]. With an increase in the content of skim milk powder, the system’s osmotic pressure rose, leading to the disruption of the cells’ structure, the leakage of the content, and the denaturation of the protein [25], resulting in a significant decrease in the viable count and lyophilization survival rate (*p* < 0.05). With 15% skim milk powder, an investigation into the addition of trehalose was conducted (Figure 1c). When the trehalose content reached 10%, the viable count of the strains (4.6 lg CFU/g) and the lyophilization survival rate (74%) were higher than those of the other levels, demonstrating the optimal protective effect. As the amount of trehalose increased, the aggregation of intracellular proteins accelerated, forming a robust glassy structure that hindered the preservation of the cells and led to poor rehydration effects, resulting in reduced survival rates. This aligns with the findings of Chen et al. [26] regarding the impact of trehalose content on the protective effect of *Lacticaseibacillus rhamnosus*. After we had determined the optimal addition levels for both protective agents, a further investigation was conducted to find the optimum proportion of the bacterial mud and protective agents, as illustrated in Figure 1d. The strain achieved the best protective effect when the ratio of bacterial mud to protective agents was 1:1.2. With an increase in the ratio, cellular permeability was affected, leading to a reduction in the viable count per unit of volume. Therefore, we selected a content of 15% skim milk powder and 10% trehalose, and a bacterial mud to protective agent ratio of 1:1.2. Under these conditions, the freeze-dried x3-2b bacterial powder exhibited a viable count of 5.1 lg CFU/g and a lyophilization survival rate of 85.58%.

### 3.2. Scanning Electron Microscopy (SEM)

**Figure 2:**
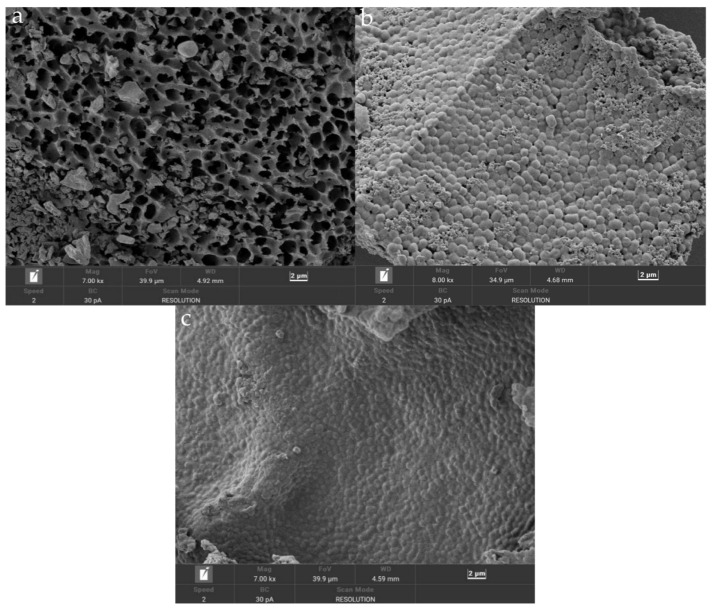
Electron microscope scanning picture of *Lactiplantibacillus plantarum* x3-2b bacteria powder. ((**a**) No preservative added; (**b**) skim milk powder added as a single protectant; (**c**) skim milk powder and trehalose added as compound protective agent).

SEM enabled the observation of the interactions between the bacterial powder and the protective agents, including phenomena such as adhesion and coverage. This supported research on the performance in terms of the stability and release of the bacterial powder. It can be seen from Figure 2a that the bacterial powder without a protective agent was seriously damaged, the gullies were obvious, and there were many holes, which may have been due to deformation or rupture of the cells and intracellular leakage of the material [26]. The individual structures of the freeze-dried bacterial powder with the addition of the single protective agent (skim milk) (Figure 2b) were distinct, and the wrapping was relatively intact. However, some pore defects were present. This indicated that the protective effect of a single protective agent on the bacterial cells was comparatively limited. In comparison, the freeze-dried bacterial powder with the addition of the composite protective agent (Figure 2c) displayed a smoother and more stable surface overall. The encapsulation of the bacteria was better, reducing the contact area between the cells and the external environment to some extent. This also isolated oxygen to prevent oxidative damage, thus maintaining the integrity of the cellular structure [27] and potentially safeguarding the bacterial cells from freeze-drying damage to a certain extent.

### 3.3. Effects of x3-2b Bacterial Powder on pH, Water Activity (a_w_), and Differences in Color of Fermented Lamb Jerky

The pH and a_w_ values are crucial indicators for assessing the degree of fermentation and the quality of fermented meat products. As shown in Table 1, the pH values of all groups decreased after fermentation, and those of the CP group and SP group were significantly lower than those of CO group (*p* < 0.05). This is due to the production of lactic acid and acidic acids by lactic acid bacteria during fermentation [28]. A relatively lower pH has a certain inhibitory effect on the growth of pathogenic and spoilage bacteria, consequently extending the shelf life of fermented lamb jerky and enhancing the effectiveness of preservation [29]. Following roasting, influenced by free amino acids and proteases, the pH values experienced a slight increase. However, the pH values of the CP group and SP group were still significantly lower than those of the CO group (*p* < 0.05). The decline in environmental pH during fermentation, approaching the isoelectric point of proteins, led to a reduction in protein’s ability to bind water [30]. After roasting, the a_w_ values of the fermented lamb jerky decreased across all groups. Notably, the CP group exhibited significantly lower a_w_ values than the CO group (*p* < 0.05). Lower a_w_ values effectively inhibited the growth of spoilage and pathogenic bacteria in fermented meat products, restrained the formation of BAs controlled by amino acid decarboxylase, and enhanced the safety of the fermented lamb jerky [31].

During the entire process, the L* values of the fermented lamb jerky in all groups exhibited an increasing trend. Moreover, the L* value of the CP group was significantly higher than that of the CO group (*p* < 0.05) (Table 1). The a* values of the CP group were consistently higher than those of the CO group, and the b* values were lower than those of the CO group. This phenomenon might be attributed to microbial facilitation of the conversion of nitrite to NO, followed by a reaction with myoglobin in the meat to produce stable nitroso myoglobin, resulting in a stable red color [32]. In this study, the E* value was introduced as the primary parameter for evaluating the color of fermented lamb jerky. After roasting, the E* value of the CP group was significantly higher than that of the CO group (*p* < 0.05), and the E* value of the SP group was lower than that of the CP group but was not significantly different (*p >* 0.05). During the roasting of fermented lamb jerky, in all groups, the E* values significantly decreased compared to the marination stage (*p* < 0.05). This phenomenon can be attributed to the elevated roasting temperature, which led to an increase in both the b* and L* values, resulting in an overall decline in the E* value. These results are consistent with the findings of Sun et al. [3], highlighting the ability of x3-2b bacterial powder prepared using composite protective agents to effectively enhance the color of fermented lamb jerky.

### 3.4. Effect of x3-2b Bacteria Powder on the Nitrite Residues in Fermented Lamb Jerky

In fermented meat products, nitrite can enhance and stabilize the color and enhance the inhibitory effect on botulinum, but the addition of excessive amounts or residues will also cause great harm to human health. Studies have shown that a lower pH can promote reductions in sodium nitrite and reduce the residual amount of nitrite [33]. As shown in Table 1, the residual nitrite levels in all groups after fermentation were lower than the initial amount (2 mg/kg) added during preparation. The nitrite residues in all fermentation groups were significantly lower than those in the CO group (*p* < 0.05). This reduction may be attributed to the x3-2b bacterial powder facilitating the conversion of nitrite to nitrate, thereby decreasing the nitrite content. After roasting, the CP group (0.431 ± 0.03 mg/kg) exhibited significantly lower residual nitrite levels than the CO group (0.654 ± 0.12 mg/kg) (*p* < 0.05). Xiao et al. [34] pointed out that microbial fermentation can decrease residual nitrite levels and inhibit the accumulation of BAs. 

### 3.5. Effect of x3-2b Bacterial Powder on Thiobarbituric Acid (TBARS) in Fermented Lamb Jerky

The oxidation of fat can have detrimental effects on the flavor and quality of fermented meat products [35]. Malondialdehyde (MDA) is one of the most common secondary products of lipid oxidation, and the TBARS value is a crucial indicator for measuring MDA [19], reflecting the extent of oxidative deterioration in products. According to Table 1, the TBARS values for all groups after marination ranged from 0 to 0.1. After fermentation, the TBA values rose in all groups, and the CP group and SP group were significantly lower than the CO group (*p* < 0.05). This trend could result from microbial oxidation and degradation contributing to lipid oxidation, consequently leading to increased TBARS values. As the roasting temperature rose, the pace of fat oxidation increased, resulting in elevated TBARS values across all groups. However, the CP group displayed significantly lower TBARS values than the SP group and CO group (*p* < 0.05). This difference could be attributed to the oxidative degradation enzymes produced by the lactic acid bacteria, which can mitigate the deterioration in the quality of fermented meat products [36]. In addition, the inclusion of trehalose in the protective agents may have contributed to the delayed oxidation of fat. The study by Castex et al. [37] demonstrated that the inclusion of lactic acid bacteria in the diet of shrimp can produce a higher overall level of antioxidants, enhancing the activity of glutathione peroxidase. In summary, the utilization of x3-2b bacterial powder prepared with composite protective agents more efficiently delayed and improved lipid oxidation, preventing excessive oxidation in fermented lamb jerky, thus enhancing the product’s quality.

### 3.6. Effect of x3-2b Bacterial Powder on the Texture of Fermented Lamb Jerky

Texture accurately reflects the compositional characteristics of food and serves as an ideal indicator for directly assessing product’s sensory qualities. As indicated in Table 2, the hardness and chewiness of the finished fermented lamb jerky were ranked as CO group > SP group > CP group. Lower values for hardness and chewiness suggest a softer texture that is easier to chew, implying that x3-2b bacterial powder contributed to the improvement in the texture of fermented lamb jerky. The hardness and chewiness of fermented lamb jerky in the CO group were significantly higher than those of the CP group (*p* < 0.05). In contrast, the elasticity and resilience of fermented lamb jerky in the CP group were significantly higher than those of the CO group (*p* < 0.05). Additionally, the cohesion of fermented lamb jerky in the CP group was significantly greater than that in the SP group and CO group (*p* < 0.05). This might be attributed to the decrease in pH after fermentation, leading to the denaturation of protein and the formation of a gel-like structure in the muscle, ultimately enhancing the hardness and elasticity of fermented lamb jerky [38].

### 3.7. Effect of x3-2b Bacterial Powder on the Biogenic Amines (BAs) in Fermented Lamb Jerky

**Figure 3:**
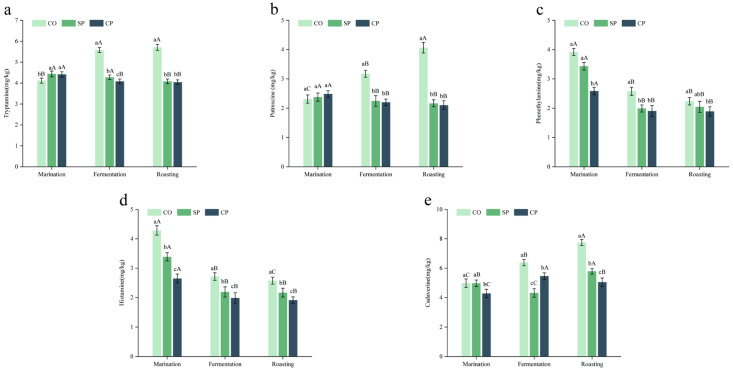
Effect of x3-2b bacterial powder on biogenic amines of fermented lamb jerky (Tryptamine (**a**); Putrescine (**b**); Phenethylamine (**c**); Histamine (**d**); Cadaverine (**e**)). ^A–C^ represents significant differences in the same group at different stages. ^a–c^ represents significant differences in different groups at the same stage (*p* < 0.05).

Tryptamine (TRY) is a type of monoamine alkaloid formed through the decarboxylation of tryptophan and the enzymatic oxidation of amines. After fermentation, the TRY content in the CP group (4.083 mg/kg) was significantly lower than that in the SP group (4.277 mg/kg) and the CO group (5.579 mg/kg) (*p* < 0.05) (Figure 3a). Even after roasting, the TRY content in the CP and SP groups remained significantly lower than that in the CO group (*p* < 0.05). Putrescine (PUT) is a polyamine present in the nucleolus, generated through the decarboxylation of ornithine and arginine. As shown in Figure 3b, after fermentation, the PUT content in the CO group was significantly higher than that in the CP group and SP group (*p* < 0.05). This could be due to the degradation of protein leading to the production of ornithine and arginine in the CO group, coupled with an increase in miscellaneous bacteria, resulting in elevated PUT levels. Following roasting, the PUT content in the CO group reached its highest level (4.065 mg/kg) (*p* < 0.05). This indicated that x3-2b bacterial powder could reduce the levels of TRY and PUT in fermented lamb jerky, which is similar to the findings of Nie et al. [39] and Bover-Cid et al. [40], who discovered that lactic acid bacteria can significantly reduce the accumulation of PUT.

Phenethylamine (PEA) is a natural compound formed through the enzyme-catalyzed decarboxylation of phenylalanine. As shown in Figure 3c, at various processing stages, the PEA content in the CP group was significantly lower than that in the CO group (*p* < 0.05). After fermentation, the PEA content in the CO group was significantly higher than that in the CP group and SP group (*p* < 0.05) (Figure 3d). After roasting, the PEA content in the SP group (2.044 mg/kg) increased slightly compared with the fermentation stage, but it was still lower than that of the CO group (2.238 mg/kg). This might be due to the increased activity of phenethylamine decarboxylase in high-temperature environments, leading to the accumulation of PEA. The PEA content in the CP group showed a declining trend across all stages, indicating that the composite protective agent provided better protection for the strains, allowing them to play a role in reducing BAs.

Histamine (HIM) is an active amino compound that acts as a neurotransmitter in the human body and can easily trigger a range of diseases, as it is very toxic to humans. As illustrated in Figure 3e, after fermentation, the HIM content of each group decreased significantly. This could be due to the good acid-producing effect of the x3-2b bacteria powder, leading to a swift reduction in the pH of the raw meat, consequently inhibiting microbial growth, and thereby decreasing the accumulation of HIM. After roasting, the HIM content in the CP group (1.914 mg/kg) was significantly lower than that in the SP group (2.618 mg/kg) and the CO group (2.574 mg/kg) (*p* < 0.05). This fell below the tolerable level for healthy individuals set by the EFSA (50 mg), indicating that the x3-2b bacterial powder prepared by the compound protective agent had an obvious effect on reducing HIM. In the process of protein denaturation, lysine decarboxylation produces cadaverine (CAD), and research has indicated that excessive CAD can react with nitrites to form nitrosamine carcinogens [41]. According to Figure 3e, after fermentation, the CAD content of the CO group was significantly higher than that of the other groups (*p* < 0.05), During the roasting stage, the CAD content continued to increase, reaching its highest value at 7.741 mg/kg, while the CAD content of CP group dropped to 5.045 mg/kg. The overall increase in CAD was due to the decomposition of lysine into CAD through the action of lysine decarboxylase, leading to a higher CAD content. In the CP group, the better protective effect of amine oxidase was obvious, which led to a smaller increase in the amount.

In conclusion, the addition of x3-2b bacterial powder reduced the content of BAs in fermented lamb jerky, and the effect was better with the x3-2b bacterial powder prepared using the composite protective agent. This might be due to the x3-2b bacterial powder reducing the proportion of amino acid decarboxylase, lowering the likelihood of the decarboxylation of biogenic amines, thereby achieving the purpose of inhibiting the formation of biogenic amines. Furthermore, it degraded the BAs already formed in the product, generated amine oxidase, and broke down BAs into less toxic substances, such as aldehydes, amines, and hydrogen peroxide, which were then transported to the surrounding cells for metabolism. Therefore, in the CP group, after the roasting of the fermented lamb jerky, the PEA content was the lowest, with significant reductions in HIM and CAD. Moreover, the degradation of other amine substances was better than that in the SP and CO groups. This indicates that the addition of x3-2b bacterial powder prepared with the composite protective agent could degrade the BAs in fermented lamb jerky.

### 3.8. Effect of x3-2b Bacteria Powder on the Volatile Flavor Compounds in Fermented Lamb Jerky

According to the analysis presented in Table 3, 70 volatile flavor substances, 18 alcohols, 13 aldehydes, 17 esters, nine acids, five ketones and eight terpenes were detected after marination, fermentation and roasting of fermented lamb jerky. Among them, there were fewer alcoholics and acids in the CO group after marination. After fermentation, the number of flavor substances in the CO and SP groups changed greatly, and the number of aldehydes and ketones decreased. After roasting, the number of esters in each group decreased, and the number of flavor substances in the CP group tended to be consistently (51) higher than that in CO group (41). These flavor compounds were generated by the degradation of carbohydrates, proteins, fats, and spices during the fermentation process [35]. 

Throughout the processing of fermented lamb jerky in each group, 2,3-butanediol emerged during fermentation; in the CP group, its content significantly increased to 14.766 μg/g after roasting. The content of α-terpineol in all groups decreased after fermentation but increased after roasting, reaching 44.063 μg/g in the CP group; both impart a clove aroma. Additionally, the content of linalool, geraniol, and citronellol within the fermented lamb jerky from the CP group was higher than that of the CO group at the roasting stage, presenting the aroma of Buddha’s hand citrus, a rose fragrance, and a lemongrass scent. Moreover, citronellol can inhibit the activity of *Staphylococcus aureus* and *Salmonella typhi*. Alcoholic compounds are usually formed through the secondary decomposition of the unsaturated fatty acids n-3 and n-6, as well as sugars, amino acids, aldehydes, or hydrogen peroxides [42]. Saturated alcohols, such as ethanol and hexanol, have higher thresholds and a minor impact on the overall flavor [43], while linalool and α-terpineol have lower flavor thresholds, enhancing the overall flavor of fermented lamb jerky [44].

Aldehyde compounds are the predominant flavor compounds in fermented lamb meat products, characterized by their low thresholds, and the oily and fruity aromas [45]. They mainly stem from the oxidation of unsaturated fatty acids (such as oleic acid, linoleic acid, arachidonic acid, and linolenic acid) [46], with their content being influenced by the activity of microbial esterases and the presence of substrates [47]. After roasting, the CP group exhibited significantly higher levels of hexanal (19.631 μg/g), heptanal (7.981 μg/g), octanal (54.457 μg/g), nonanal (125.925 μg/g), and decanal (24.875 μg/g) compared to the other stages and groups (*p* < 0.05). These aldehyde compounds contributed distinct aromas, including natural grassy, barbecue, fruity jasmine, intense oily, and sweet orange fragrances. Additionally, the CP group of fermented lamb jerky contained a significant amount of myristic aldehyde (148.533 μg/g), which imparts a strong peach aroma. These low-threshold, rapidly oxidizing flavor compounds contributed substantially to the overall flavor of fermented lamb jerky [48].

Acidic compounds primarily include the organic acids produced by microorganisms during fermentation, which serve as the main source of fruity aromas in fermented meat products [19]. After fermentation, the acetic acid content in the SP and CP groups exceeded that of the CO group, indicating the obvious acid-producing characteristics of the x3-2b bacterial powder. Additionally, the acetic acid levels were consistently higher in the CP group than in the SP group at all stages, indicating better protection against bacteria. After roasting, acetic acid imparts a vinegar-like flavor to fermented lamb jerky.

Aldehydes undergo oxidation to form alcohols and acids, and subsequently, through esterification, produce ester compounds [49]. Ester substances are mainly present after the marination and fermentation stages. However, high-temperature processing is unfavorable for the formation of ester compounds, resulting in their reduction after roasting [50]. After roasting, the CP group exhibited significant levels of methyl valerate (31.170 μg/g) and bornyl acetate (11.719 μg/g), making notable flavor contributions characterized by a pine aroma. Additionally, the content of these compounds in the CP group exceeded that of the SP and CO groups. Terpene compounds are predominantly derived from spices. After roasting stage of the CP group, 3-carene (16.917 μg/g) had a lemony fruity fragrance, while β-caryophyllene (18.144 μg/g) and α-caryophyllene (18.396 μg/g) lent a delicate clove aroma, making a certain contribution to the overall flavor.

Ketone compounds are formed through the oxidation of unsaturated fatty acids [51] and microbial oxidation reactions [52]. After roasting, 3-hydroxy-2-butanone (12.692 μg/g) was detected in the CP group, significantly surpassing the level in the other two groups (*p* < 0.05). This compound is produced through the conversion of pyruvate, which is generated by the metabolism of glucose in meat and imparts a milky aroma. Additionally, after roasting, methyl heptenone (8.837 μg/g) and methyl nonyl ketone (38.912 μg/g) were identified in the CP group, offering fruity/fresh, and citrus aromas, respectively, thus greatly contributing to the overall flavor of the fermented lamb jerky. Consequently, the more comprehensive protection of bacterial cells by the composite protective agent enabled lactic acid bacteria to enhance the variety and content of compounds through metabolism, giving fermented lamb jerky its distinct flavor.

## 4. Conclusions

In this study, the optimal formulation of freeze-dried protective agent, including 15% skim milk powder and 10% trehalose was used to prepare freeze-dried x3-2b bacterial powder with a ratio of bacterial mud to protective agent of 1:1.2, which improved the survival rate of strains during the freeze-drying process. Through electron microscopy, it was found that the surface morphology of the freeze-dried bacterial cells became smoother with the addition of protective agents. Additionally, the protective agents exhibited good wrapping around the cells, thereby reducing the cellular damage caused by the formation of ice crystals. To enhance the value of x3-2b bacterial powder, it was used as a fermenting agent in the production of fermented lamb jerky. The results indicated that inoculation with the x3-2b bacterial powder prepared using a composite protective agent could accelerate the reduction in pH and a_w_ during the fermentation of lamb jerky. This led to improvements in the color and texture of the fermented lamb jerky, as well as an increase in the variety and content of flavor compounds such as alcoholics, aldehydes, acids, and terpenes. Additionally, there was a noticeable reduction in the levels of PEA, HIM, and CAD in the fermented lamb jerky. The inhibition of other amine substances was superior to that of the CO group and SP group, thereby reducing the accumulation of BAs in the fermented lamb jerky.

## Figures and Tables

**Table 1:** The effect of x3-2b bacterial powder on the physicochemical quality of fermented lamb jerky.

		CO	SP	CP
pH	Marination	5.64 ± 0.02 ^Aa^	5.58 ± 0.02 ^Ab^	5.55 ± 0.01 ^Ac^
	Fermentation	5.34 ± 0.02 ^Ca^	4.77 ± 0.02 ^Cb^	4.74 ± 0.01 ^Bb^
	Roasting	5.51 ± 0.01 ^Ba^	4.83 ± 0.01 ^Bb^	4.78 ± 0.02 ^Bc^
a_w_	Marination	0.887 ± 0.015 ^Aa^	0.861 ± 0.060 ^Ab^	0.859 ± 0.010 ^Ab^
	Fermentation	0.835 ± 0.045 ^Ba^	0.776 ± 0.120 ^Bb^	0.780 ± 0.100 ^Bb^
	Roasting	0.615 ± 0.015 ^Ca^	0.594 ± 0.002 ^Cb^	0.587 ± 0.001 ^Cb^
Luminance value L*	Marination	39.60 ± 0.08 ^Bb^	41.04 ± 0.67 ^Cb^	44.37 ± 0.22 ^Ba^
	Fermentation	36.55 ± 0.37 ^Cb^	45.23 ± 0.06 ^Ba^	45.32 ± 0.18 ^Ba^
	Roasting	45.41 ± 1.35 ^Ab^	48.29 ± 1.59 ^Aa^	48.72 ± 2.23 ^Aa^
Red degree value a*	Marination	14.77 ± 0.17 ^Ac^	15.20 ± 0.27 ^Ab^	16.98 ± 0.19 ^Aa^
	Fermentation	9.02 ± 0.16 ^Cb^	10.46 ± 0.13 ^Ca^	10.79 ± 0.15 ^Ca^
	Roasting	10.91 ± 0.17 ^Bc^	11.50 ± 0.11 ^Bb^	12.09 ± 0.16 ^Ba^
Yellowness value b*	Marination	10.72 ± 0.16 ^Ba^	10.39 ± 0.17 ^Ba^	10.21 ± 0.22 ^Ba^
	Fermentation	10.67 ± 0.11 ^Ba^	9.59 ± 0.15 ^Cb^	8.86 ± 0.17 ^Cc^
	Roasting	14.42 ± 0.19 ^Aa^	13.88 ± 0.35 ^Ab^	13.67 ± 0.61 ^Ab^
E* values	Marination	1.85 ± 0.03 ^Aa^	1.87 ± 0.03 ^Aa^	1.90 ± 0.02 ^Aa^
	Fermentation	1.87 ± 0.05 ^Ab^	1.90 ± 0.02 ^Aab^	1.95 ± 0.03 ^Aa^
	Roasting	1.01 ± 0.02 ^Bb^	1.07 ± 0.02 ^Bab^	1.12 ± 0.03 ^Ba^
Nitrite contents (mg/kg)	Marination	0.555 ± 0.30 ^Ca^	0.255 ± 0.06 ^Cab^	0.108 ± 0.03 ^Bb^
	Fermentation	0.737 ± 0.19 ^Aa^	0.601 ± 0.06 ^Ab^	0.489 ± 0.14 ^Ac^
	Roasting	0.654 ± 0.12 ^Ba^	0.490 ± 0.26 ^Bab^	0.431 ± 0.03 ^Ab^
TBARS content (mg/100 g)	Marination	0.08 ± 0.03 ^Ba^	0.05 ± 0.03 ^Bb^	0.10 ± 0.02 ^Aa^
	Fermentation	0.16 ± 0.01 ^Aa^	0.11 ± 0.01 ^Ab^	0.13 ± 0.01 ^Ab^
	Roasting	0.20 ± 0.02 ^Aa^	0.12 ± 0.01 ^Ab^	0.06 ± 0.02 ^Bc^

^A–C^: Mean values followed by different uppercase letter in the same column indicate significant difference. ^a–c^: Mean values followed by different lowercase letters in the same row indicate significant difference (*p* < 0.05). CO: the lamb jerky not inoculated with the fermentation agent served; SP: x3-2b bacteria powder prepared with skimmed milk powder added as a single protective agent; CP: x3-2b bacteria powder prepared with skimmed milk powder and trehalose added as compound protective agent.

**Table 2:** Effect of x3-2b bacterial powder on dry texture of finished fermented lamb jerky.

	CO	SP	CP
Hardness (N)	4426.14 ± 660.75 ^a^	2765.35 ± 338.33 ^b^	2515.31 ± 99.35 ^b^
Elasticity	0.55 ± 0.01 ^b^	0.60 ± 0.04 ^ab^	0.68 ± 0.10 ^a^
Cohesion	0.52 ± 0.05 ^b^	0.55 ± 0.02 ^b^	0.66 ± 0.04 ^a^
Stickiness	2474.58 ± 643.59 ^a^	1102.36 ± 110.45 ^b^	896.41 ± 75.02 ^c^
Chewiness (N)	1357.84 ± 328.83 ^a^	750.63 ± 112.20 ^b^	541.79 ± 57.37 ^b^
Resilience	0.12 ± 0.02 ^c^	0.18 ± 0.02 ^b^	0.22 ± 0.01 ^a^

^a–c^: Mean values followed different lowercase letters in the same row indicate significant difference (*p* < 0.05). CO: the lamb jerky not inoculated with the fermentation agent served; SP: x3-2b bacteria powder prepared with skimmed milk powder added as a single protective agent; CP: x3-2b bacteria powder prepared with skimmed milk powder and trehalose added as compound protective agent.

**Table 3:** Changes of volatile flavor substances during processing of fermented lamb jerky (μg/g).

Volatile Compound	Chemical Formula	Groups	Stage		
			Marination	Fermentation	Roasting
**Alcohols (18)**					
2,3-Butanediol	C_4_H_10_O_2_	CO	—	6.438 ± 0.294	8.668 ± 4.330
		SP	—	4.015 ± 0.398	1.747 ± 0.786
		CP	—	4.451 ± 0.535	14.766 ± 6.935
1,3-Butanediol	C_4_H_10_O_2_	CO	—	—	—
		SP	—	—	1.374 ± 0.000
		CP	—	—	2.221 ± 0.000
Erythritol	C_4_H_10_O_4_	CO	—	—	11.459 ± 0.000
		SP	—	2.374 ± 0.000	—
		CP	—	—	3.663 ± 0.000
2-Heptanol	C_7_H_16_O	CO	2.001 ± 0.000	—	—
		SP	0.157 ± 0.005	—	—
		CP	0.230 ± 0.020	—	—
Trans-2-octen-1-ol	C_8_H_16_O	CO	—	—	0.358 ± 0.006
		SP	0.367 ± 0.000	0.377 ± 0.109	0.360 ± 0.000
		CP	0.377 ± 0.000	0.386 ± 0.000	2.304 ± 0.348
1-Octen-3-ol	C_8_H_16_O	CO	—	—	—
		SP	0.291 ± 0.022	—	1.037 ± 0.042
		CP	0.305 ± 0.000	—	1.529 ± 0.011
2-Propyl-1-pentanol	C_8_H_18_O	CO	—	24.730 ± 0.000	18.835 ± 3.382
		SP	—	2.063 ± 0.027	11.757 ± 5.062
		CP	—	12.056 ± 0.000	12.870 ± 8.692
N-octanol	C_8_H_18_O	CO	—	0.694 ± 0.000	2.072 ± 0.471
		SP	0.734 ± 0.000	0.706 ± 0.137	2.848 ± 0.000
		CP	—	0.977 ± 0.110	—
Verbenol	C_10_H_16_O	CO	—	0.398 ± 0.000	—
		SP	0.057 ± 0.000	1.162 ± 0.000	—
		CP	0.095 ± 0.000	—	—
Linalool	C_10_H_18_O	CO	6.717 ± 2.406 ^a^	2.185 ± 1.46	8.193 ± 1.513
		SP	6.204 ± 1.937 ^ab^	2.932 ± 0.186	7.946 ± 1.693
		CP	6.281 ± 0.058 ^b^	6.164 ± 1.994	64.220 ± 6.461
Isopulegol	C_10_H_18_O	CO	0.871 ± 0.000	—	—
		SP	0.118 ± 0.039	—	—
		CP	0.140 ± 0.052	—	0.806 ± 0.000
α-terpineol	C_10_H_18_O	CO	8.599 ± 2.804	4.058 ± 1.051	5.187 ± 2.285
		SP	5.205 ± 3.436	5.692 ± 0.000	6.947 ± 1.789
		CP	7.707 ± 0.000	6.867 ± 2.545	44.063 ± 4.509
Geraniol	C_10_H_18_O	CO	1.841 ± 0.012	1.646 ± 0.000	2.212 ± 0.585
		SP	2.078 ± 0.274	1.287 ± 0.859	3.873 ± 0.000
		CP	—	1.133 ± 0.000	14.191 ± 0.000
DL-isoborneol	C_10_H_18_O	CO	—	0.174 ± 0.000	0.486 ± 0.045
		SP	—	0.161 ± 0.004	0.460 ± 0.000
		CP	—	0.249 ± 0.031	—
Nerol	C_10_H_18_O	CO	—	—	—
		SP	4.188 ± 0.000	0.607 ± 0.212	—
		CP	1.681 ± 1.156	2.324 ± 0.000	13.498 ± 0.000
Citronellol	C_10_H_20_O	CO	6.871 ± 0.638	1.175 ± 0.018	1.249 ± 0.945
		SP	1.909 ± 0.667	1.108 ± 0.256	3.182 ± 0.000
		CP	2.038 ± 0.141	1.105 ± 0.008	12.484 ± 0.000
Trans-Nerolidol	C_15_H_26_O	CO	3.946 ± 0.254	0.749 ± 0.000	1.011 ± 0.372
		SP	2.059 ± 0.346	—	1.765 ± 0.785
		CP	1.417 ± 0.289	0.786 ± 0.000	5.621 ± 0.000
2-hexadecanol	C_16_H_34_O	CO	0.325 ± 0.000	0.327 ± 0.240	0.131 ± 0.077
		SP	0.408 ± 0.000	2.603 ± 0.383	—
		CP	0.079 ± 0.000	0.056 ± 0.000	—
**Aldehydes (13)**					
Succinaldehyde	C_4_H_6_O_2_	CO	0.123 ± 0.040	—	—
		SP	—	—	2.004 ± 0.451
		CP	0.419 ± 0.000	—	1.065 ± 0.296
3-Butanolal	C_4_H_8_O_2_	CO	0.181 ± 0.000	—	—
		SP	0.110 ± 0.002	—	—
		CP	0.502 ± 0.000	0.222 ± 0.000	0.916 ± 0.000
Isovaleraldehyde	C_5_H_10_O	CO	—	—	1.565 ± 0.741
		SP	—	—	0.684 ± 2.677
		CP	—	—	19.431 ± 1.028
Valeraldehyde	C_5_H_10_O	CO	—	—	—
		SP	0.103 ± 0.000	—	0.053 ± 0.000
		CP	—	0.047 ± 0.000	0.118 ± 0.042
Hexanal	C_6_H_12_O	CO	1.657 ± 0.431 ^Ba^	0.424 ± 0.198 ^Cc^	2.813 ± 0.450 ^Ab^
		SP	0.719 ± 0.020 ^Cb^	1.726 ± 0.053 ^Ba^	4.031 ± 0.010 ^Ab^
		CP	0.284 ± 0.150 ^Bc^	0.973 ± 0.193 ^Bb^	19.631 ± 3.991 ^Aa^
Heptaldehyde	C_7_H_14_O	CO	0.841 ± 0.700 ^Ba^	0.408 ± 0.158 ^Cb^	3.226 ± 0.947 ^Ab^
		SP	0.197 ± 0.083 ^Cb^	4.015 ± 0.038 ^Aa^	3.467 ± 0.701 ^Bb^
		CP	0.146 ± 0.031 ^Bb^	0.351 ± 0.108 ^Bb^	7.981 ± 0.942 ^Aa^
(E)-2-Octenal	C_8_H_14_O	CO	1.887 ± 0.000	—	0.581 ± 0.132
		SP	—	1.051 ± 0.309	0.942 ± 0.606
		CP	—	1.699 ± 0.886	6.075 ± 0.217
Octanal	C_8_H_16_O	CO	4.092 ± 3.439 ^Ba^	2.562 ± 1.558 ^Ca^	13.763 ± 3.318 ^Ab^
		SP	0.541 ± 0.113 ^Cb^	1.834 ± 0.329 ^Bb^	9.471 ± 0.000 ^Ac^
		CP	0.660 ± 0.082 ^Cb^	2.136 ± 0.318 ^Ba^	54.457 ± 0.007 ^Aa^
Trans-2-nonenal	C_9_H_16_O	CO	0.882 ± 0.757	1.538 ± 0.000	1.222 ± 0.227
		SP	0.161 ± 0.035	0.528 ± 0.227	—
		CP	0.151 ± 0.029	0.733 ± 0.049	7.060 ± 0.000
2-Nonenal	C_9_H_16_O	CO	—	0.400 ± 0.000	—
		SP	—	—	1.239 ± 0.000
		CP	—	3.702 ± 4.250	10.009 ± 0.000
Nonanal	C_9_H_18_O	CO	26.055 ± 2.090 ^Ba^	19.357 ± 2.643 ^Ca^	37.476 ± 1.749 ^Ac^
		SP	4.283 ± 1.370 ^Bb^	5.463 ± 1.225 ^Bb^	42.677 ± 2.291 ^Ab^
		CP	3.485 ± 0.341 ^Bb^	4.569 ± 2.429 ^Bb^	125.925 ± 6.280 ^Aa^
Decanal	C_10_H_20_O	CO	0.349 ± 0.357 ^Bb^	0.321 ± 0.796 ^Bc^	3.981 ± 0.682 ^Ab^
		SP	0.708 ± 0.021 ^Ba^	0.676 ± 0.339 ^Bb^	4.898 ± 1.937 ^Ab^
		CP	0.854 ± 0.127 ^Ca^	1.431 ± 0.029 ^Ba^	24.875 ± 6.073 ^Aa^
Myristic aldehyde	C_14_H_28_O	CO	1.840 ± 4.747	1.683 ± 0.435	15.905 ± 4.565
		SP	1.766 ± 0.291	1.778 ± 0.192	37.378 ± 6.599
		CP	1.866 ± 0.568	3.535 ± 0.842	148.533 ± 4.09
**Esters (17)**					
Diethyl ethylene	C_6_H_10_O_4_	CO	—	—	3.365 ± 0.372
		SP	—	—	2.689 ± 0.397
		CP	—	—	2.608 ± 2.712
Methyl valerate	C_6_H_12_O_2_	CO	5.356 ± 4.488 ^Aa^	0.719 ± 0.000 ^Bc^	5.074 ± 0.112 ^Ab^
		SP	0.594 ± 0.187 ^Cb^	2.555 ± 0.651 ^Bb^	4.199 ± 0.000 ^Ab^
		CP	0.580 ± 0.085 ^Cb^	3.426 ± 0.121 ^Ba^	31.170 ± 4.141 ^Aa^
Ethyl valerate	C_7_H_14_O_2_	CO	0.829 ± 0.691	—	—
		SP	—	1.078 ± 0.128	—
		CP	0.186 ± 0.010	2.110 ± 0.136	—
Methyl hexanoate	C_7_H_14_O_2_	CO	5.289 ± 0.809	1.150 ± 0.100	—
		SP	0.639 ± 0.225	0.240 ± 0.000	—
		CP	0.470 ± 0.176	0.696 ± 0.000	—
Ethyl Hexanoate	C_8_H_16_O_2_	CO	3.082 ± 0.597	—	—
		SP	0.650 ± 0.209	—	—
		CP	0.578 ± 0.100	1.112 ± 0.000	—
Caprylic acid methyl ester	C_9_H_18_O_2_	CO	14.036 ± 1.870	2.686 ± 0.000	—
		SP	1.345 ± 0.171	0.680 ± 0.134	—
		CP	1.337 ± 0.217	0.482 ± 0.045	—
Ethyl caprylate	C_10_H_20_O_2_	CO	5.227 ± 0.433	—	—
		SP	1.010 ± 0.269	0.678 ± 0.000	—
		CP	0.990 ± 0.205	0.893 ± 0.381	—
Methyl nonanoate	C_10_H_20_O_2_	CO	2.273 ± 0.907	0.412 ± 0.234	—
		SP	0.420 ± 0.062	0.181 ± 0.009	—
		CP	0.406 ± 0.073	—	—
Geranyl formate	C_11_H_18_O_2_	CO	—	—	—
		SP	0.319 ± 0.067	0.208 ± 0.000	—
		CP	—	0.689 ± 0.000	—
Methyl decanoate	C_11_H_22_O_2_	CO	6.717 ± 0.569	1.787 ± 0.079	—
		SP	1.011 ± 0.131	0.448 ± 0.134	—
		CP	1.217 ± 0.240	0.391 ± 0.029	—
Linalyl acetate	C_12_H_20_O_2_	CO	0.143 ± 0.000	0.177 ± 0.000	0.636 ± 0.157
		SP	0.227 ± 0.059	0.224 ± 0.000	0.766 ± 0.337
		CP	0.215 ± 0.050	2.342 ± 0.000	3.701 ± 0.646
Bornyl acetate	C_12_H_20_O_2_	CO	1.711 ± 0.467 ^Aa^	0.370 ± 0.055^Cc^	1.431 ± 0.350 ^Bb^
		SP	0.669 ± 0.269 ^Ab^	0.447 ± 0.148 ^Bb^	—
		CP	—	0.778 ± 0.096 ^Ba^	11.719 ± 1.612 ^Aa^
Ethyl caprate	C_12_H_24_O_2_	CO	1.008 ± 0.827	0.753 ± 0.374	—
		SP	—	0.176 ± 0.029	—
		CP	0.369 ± 0.100	0.272 ± 0.077	—
Methyl laurate	C_13_H_26_O_2_	CO	6.549 ± 0.344	1.627 ± 0.000	—
		SP	1.330 ± 0.239	—	—
		CP	2.252 ± 0.000	—	—
Methyl myristate	C_15_H_30_O_2_	CO	3.723 ± 0.859	1.651 ± 0.493	—
		SP	1.024 ± 0.123	0.421 ± 0.094	0.256 ± 0.014
		CP	1.165 ± 0.225	0.418 ± 0.109	—
Methyl palmitate	C_17_H_34_O_2_	CO	7.857 ± 0.000	1.347 ± 0.409	0.177 ± 0.053
		SP	1.506 ± 0.099	0.684 ± 0.023	0.323 ± 0.001
		CP	1.671 ± 0.472	0.205 ± 0.060	0.583 ± 0.000
Ethyl palmitate	C_18_H_36_O_2_	CO	1.614 ± 0.312	0.979 ± 0.442	0.130 ± 0.012
		SP	0.391 ± 0.118	—	0.161 ± 0.057
		CP	0.605 ± 0.112	0.251 ± 0.027	0.583 ± 0.000
**Acids (9)**					
L-alanylglycine	C_5_H_10_N_2_O_3_	CO	0.163 ± 0.000	0.613 ± 0.337	0.925 ± 0.509
		SP	0.256 ± 0.141	0.314 ± 0.000	0.202 ± 0.061
		CP	0.270 ± 0.063	—	5.486 ± 0.468
Valeric acid	C_5_H_10_O_2_	CO	—	—	0.713 ± 0.156
		SP	0.179 ± 0.141	0.394 ± 0.000	8.747 ± 0.343
		CP	—	0.248 ± 0.010	6.857 ± 0.381
Acetic acid	C_8_H_16_O_4_	CO	5.168 ± 0.000	2.791 ± 0.988	26.559 ± 4.280
		SP	0.230 ± 0.000	17.207 ± 0.048	16.520 ± 1.031
		CP	—	19.976 ± 0.354	17.162 ± 1.348
Hydrocinnamic acid	C_9_H_10_O_2_	CO	51.837 ± 0.000	9.304 ± 0.000	—
		SP	0.055 ± 0.000	9.103 ± 0.748	8.775 ± 0.401
		CP	0.274 ± 0.000	5.686 ± 0.309	1.626 ± 0.488
2-Undecenoic acid	C_11_H_20_O_2_	CO	—	—	—
		SP	—	—	—
		CP	0.193 ± 0.000	—	3.192 ± 0.254
3-Hydroxydodecanoic acid	C_12_H_24_O_3_	CO	0.162 ± 0.131	0.229 ± 0.075	—
		SP	0.062 ± 0.009	0.319 ± 0.241	4.375 ± 0.151
		CP	—	0.150 ± 0.037	5.103 ± 0.000
17-octadecynoic acid	C_18_H_32_O_2_	CO	0.208 ± 0.000	—	0.397 ± 0.048
		SP	—	—	0.434 ± 0.322
		CP	0.127 ± 0.000	1.035 ± 0.000	2.993 ± 0.000
Oleic acid	C_18_H_34_O_2_	CO	—	0.433 ± 0.370	—
		SP	0.137 ± 0.079	0.604 ± 0.000	0.569 ± 0.000
		CP	0.340 ± 0.229	0.558 ± 0.388	—
Trans-13-Octadecenoic acid	C_18_H_34_O_2_	CO	—	—	—
		SP	—	—	—
		CP	—	1.567 ± 0.000	0.923 ± 0.000
**Ketones (5)**					
3-Hydroxy-2-butanone	C_4_H_8_O_2_	CO	2.775 ± 0.000	3.693 ± 0.553	7.389 ± 0.488 ^c^
		SP	—	0.309 ± 0.030	0.265 ± 0.000 ^b^
		CP	—	0.499 ± 0.414	12.692 ± 7.241 ^a^
Methyl heptenone	C_8_H_14_O	CO	0.956 ± 0.000	0.315 ± 0.128	1.077 ± 0.402
		SP	0.376 ± 0.136	0.148 ± 0.000	1.190 ± 0.258
		CP	0.279 ± 0.045	0.336 ± 0.156	8.837 ± 0.656
4-Octanone	C_8_H_16_O	CO	3.174 ± 0.907	2.645 ± 0.032	2.779 ± 0.212
		SP	3.220 ± 0.239	2.950 ± 0.000	2.863 ± 0.164
		CP	3.284 ± 0.483	2.549 ± 0.195	8.678 ± 0.760
Piperitone	C_10_H_16_O	CO	1.881 ± 0.535	—	—
		SP	0.450 ± 0.148	0.216 ± 0.000	—
		CP	0.448 ± 0.080	0.362 ± 0.000	4.568 ± 0.719
Methyl nonyl ketone	C_11_H_22_O	CO	7.353 ± 0.205	—	4.329 ± 0.036
		SP	2.213 ± 0.531	—	6.189 ± 0.184
		CP	1.934 ± 0.357	2.561 ± 0.180	38.912 ± 0.646
**Terpenes (8)**					
2,4-Diemthylstyrene	C_10_H_12_	CO	—	—	—
		SP	0.413 ± 0.000	—	—
		CP	0.743 ± 0.048	—	—
Cis-Anethol	C_10_H_12_O	CO	1.113 ± 0.000	1.020 ± 0.302	2.362 ± 0.610
		SP	1.835 ± 0.538	0.898 ± 0.201	4.333 ± 0.000
		CP	1.777 ± 0.449	1.670 ± 0.397	15.919 ± 0.000
3-Carene	C_10_H_16_	CO	0.377 ± 0.000	0.200 ± 0.000	2.743 ± 0.754
		SP	0.065 ± 0.025	0.102 ± 0.000	4.175 ± 0.101
		CP	0.053 ± 0.001	0.567 ± 0.000	16.917 ± 4.787
α-Pinene	C_15_H_24_	CO	1.615 ± 0.377	—	—
		SP	0.480 ± 0.131	—	3.719 ± 0.000
		CP	—	—	27.814 ± 0.657
β-Caryophyllene	C_15_H_24_	CO	19.801 ± 1.683	4.539 ± 0.468	24.177 ± 6.468
		SP	4.675 ± 3.611	6.724 ± 3.234	14.050 ± 4.725
		CP	7.214 ± 1.140	8.555 ± 1.112	18.144 ± 4.937
α-caryophyllene	C_15_H_24_	CO	3.606 ± 0.000	0.711 ± 0.000	1.199 ± 0.255
		SP	0.696 ± 0.213	0.585 ± 0.213	2.243 ± 0.765
		CP	0.773 ± 0.113	0.756 ± 0.096	18.396 ± 2.725
Cedrene	C_15_H_24_	CO	35.454 ± 29.518	0.681 ± 0.511	9.485 ± 13.019
		SP	7.552 ± 5.869	0.447 ± 0.000	2.094 ± 1.475
		CP	5.030 ± 4.800	—	—
Caryophylleneoxide	C_15_H_24_O	CO	0.857 ± 0.706	0.179 ± 0.000	0.578 ± 0.439
		SP	1.244 ± 0.000	0.585 ± 0.213	0.826 ± 0.633
		CP	—	0.164 ± 0.000	4.134 ± 1.702

^A–C^: Mean values followed by different uppercase letter in the same row indicate significant difference. ^a–c^: Mean values followed by different lowercase letters in the same column indicate significant difference (*p* < 0.05). CO: the lamb jerky not inoculated with the fermentation agent served; SP: x3-2b bacteria powder prepared with skimmed milk powder added as a single protective agent; CP: x3-2b bacteria powder prepared with skimmed milk powder and trehalose added as compound protective agent.

## Data Availability

The data used to support the findings of this study can be made available by the corresponding author upon request.
